# Dietary Protein, Fiber and Coffee Are Associated with Small Intestine Microbiome Composition and Diversity in Patients with Liver Cirrhosis

**DOI:** 10.3390/nu12051395

**Published:** 2020-05-13

**Authors:** Shehnaz K. Hussain, Tien S. Dong, Vatche Agopian, Joseph R. Pisegna, Francisco A. Durazo, Pedram Enayati, Vinay Sundaram, Jihane N. Benhammou, Mazen Noureddin, Gina Choi, Walid S. Ayoub, Venu Lagishetty, David Elashoff, Marc T. Goodman, Jonathan P. Jacobs

**Affiliations:** 1 Cedars-Sinai Cancer and Department of Medicine, Cedars-Sinai Medical Center, Los Angeles, CA 90048, USA; Marc.Goodman@cshs.org; 2 The Vatche and Tamar Manoukian Division of Digestive Diseases, Department of Medicine, David Geffen School of Medicine at University of California, Los Angeles, CA 90095, USA; TSDong@mednet.ucla.edu (T.S.D.); FDurazo@mednet.ucla.edu (F.A.D.); JJacobs@mednet.ucla.edu (J.P.J.); 3 UCLA Microbiome Center, David Geffen School of Medicine at University of California, Los Angeles, CA 90095, USA; VLagishetty@mednet.ucla.edu; 4 Department of Surgery, University of California, Los Angeles, CA 90095, USA; VAgopian@mednet.ucla.edu (V.A.); JBenhammou@mednet.ucla.edu (J.N.B.); GinaChoi@mednet.ucla.edu (G.C.); 5 Division of Gastroenterology, Hepatology and Parenteral Nutrition, Veterans Administration Greater Los Angeles Healthcare System, Los Angeles, CA 90073, USA; JPisegna@mednet.ucla.edu; 6 Department of Medicine and Human Genetics, David Geffen School of Medicine at University of California, Los Angeles, CA 90095, USA; 7 Division of Digestive and Liver Diseases, Department of Medicine, Cedars-Sinai Medical Center, Los Angeles, CA 90048, USA; Pedram.Enayati@cshs.org (P.E.); Vinay.Sundaram@cshs.org (V.S.); Mazen.Noureddin@cshs.org (M.N.); Walid.Ayoub@cshs.org (W.S.A.); 8 Department of Medicine, University of California, Los Angeles, CA 90095, USA; DElashoff@mednet.ucla.edu; 9 Department of Biostatistics, Fielding School of Public Health, University of California, Los Angeles, CA 90095, USA

**Keywords:** liver cirrhosis, duodenal microbiome, diet

## Abstract

The gut microbiome is a key factor in chronic liver disease progression. In prior research, we found that the duodenal microbiome was associated with sex, ethnicity, and cirrhosis complications. Here, we examined the association between diet and the duodenal microbiome in patients with liver cirrhosis. This study included 51 participants who completed a detailed food frequency questionnaire and donated duodenal biopsies for microbiome characterization by 16S ribosomal RNA gene sequencing. Data were analyzed for alpha diversity, beta diversity, and association of taxa abundance with diet quality and components using QIIME 2 pipelines. Diet quality was assessed through calculation of the Healthy Eating Index 2010. Participants with higher adherence to protein recommendations exhibited increased microbial richness and evenness (*p* = 0.03) and a different microbial profile compared to those with lower adherence (*p* = 0.03). *Prevotella-9* and *Agathobacter* were increased in association with increased protein adherence. Fiber consumption was also associated with the duodenal microbial profile (*p* = 0.01), with several taxa exhibiting significantly decreased or increased abundance in association with fiber intake. Coffee drinking was associated with microbial richness and evenness (*p* = 0.001), and there was a dose–response association between coffee drinking and relative abundance of *Veillonella* (*p* = 0.01). We conclude that protein, fiber, and coffee are associated with diversity and composition of the duodenal microbiome in liver cirrhosis.

## 1. Introduction

Liver cirrhosis is characterized by an irreversible state of hepatic injury that includes vascular distortion, fibrosis, and inflammation, and is considered the penultimate step of the multistage pathogenesis of hepatocellular carcinoma (HCC). HCC is a highly lethal cancer with rising incidence and mortality rates [1,2]. Latinos carry a heavy burden, and also appear to have a more rapid progression from cirrhosis to HCC compared to other major racial/ethnic groups [2,3]. U.S.-born Latinos, particularly male adults, are at greater risk of HCC than foreign-born Latinos, suggesting an adverse acculturation effect that has been attributed in part to changes in dietary habits [2].

A picture is beginning to emerge regarding how imbalances in the gut–liver axis influence the progression from cirrhosis to HCC [4,5]. The portal venous system, which carries nutrients, bacterial components and metabolites from the intestine is the principle circulation to the liver. Liver exposure to these highly immunogenic features is increased when gut barrier function is compromised, as it is in cirrhosis [6]. Specifically, there is a growing body of literature regarding the hepatocarcinogenic potential of the secondary bile acid deoxycholic acid (DCA), which is a metabolic product of resident intestinal microbes, via its ability to activate immune cells, induce cytokine secretion, and promote hepatocyte damage and transformation [7,8]. Additionally, lipopolysaccharide (LPS, a potent immunogenic component of Gram-negative bacterial cell membranes) translocation, is emerging as a key factor in the pathogenesis of HCC [9,10,11,12].

Additionally, cirrhosis is associated with dysbiosis, which is also thought to influence HCC pathogenesis. The relative abundance of potentially pathogenic taxa increases, while beneficial bacterial abundance decreases, as liver disease worsens [13,14]. In addition to these compositional changes, functional changes, including increased LPS and altered bile acid compositions, have also been reported in association with progressive disease states [15]. Furthermore, recent epidemiological studies have reported associations between intestinal dysbiosis and liver cancer [16,17,18], suggesting that the microbiome may be a biomarker for perturbations in the gut–liver axis during the early stages of HCC.

It is now understood that diet plays a significant role in shaping the digestive tract microbiome, although much remains to be learned regarding the influence of specific dietary features including temporal patterns, individual nutrients, whole foods, and diet quality [19,20,21]. Thus, to understand the influence of the microbiome in HCC pathogenesis, it is necessary to characterize the association of diet with the microbiome in the setting of cirrhosis. Importantly, prior studies demonstrate that diet is a key risk factor for cirrhosis progression and development of HCC [22]. Of particular importance appears to be protein adequacy and animal protein [22,23,24,25], whole grains, particularly fiber [26,27,28], and coffee [29], with emerging evidence to suggest that the microbiome mediates the effects of these diet components on liver disease and cancer [30]. Foods are eaten in combination, creating a challenge in distinguishing the influence of individual food items in standard single food- or nutrient-based analysis, which can be overcome with dietary pattern-based analysis. Diet quality indexes (DQI), such as the Healthy Eating Index-2010 (HEI-2010), capture the complexity of the entire diet. Higher DQI scores reflect a positive adherence to a healthful diet. In prior research, high DQI was associated with lower HCC incidence and liver disease mortality [31].

In this study, we examined the association of specific dietary habits selected a priori (overall diet quality, protein, whole grains, fiber, and coffee consumption) with microbiome diversity and composition in patients with liver cirrhosis at risk of HCC. In view of the critical role played by the small intestine in digestion through hormone secretion, absorption, bile acid metabolism, and development of mucosal immunity, we have focused our efforts here to characterize the small intestine microbiome.

## 2. Methods

### 2.1. Study Population

We conducted a cross-sectional study including 51 participants with liver cirrhosis enrolled in an ongoing prospective cohort study designed to uncover biomarkers and pathways for development of HCC, called the Microbiome, Microbial Markers, and Liver Disease (M_3_LD) Study [32]. Patients with a diagnosis of liver cirrhosis confirmed by biopsy or imaging, who were 18 years of age or older, were eligible for the study. Having a prior organ transplant or diagnosis of HCC were the only exclusion criteria. We kept the inclusion broad in order to most closely represent the underlying population of patients with liver cirrhosis. Participants were recruited from three medical centers in southern California: Cedars-Sinai Medical Center (CSMC), Ronald Reagan UCLA Medical Center, and Veterans Affairs Greater Los Angeles Healthcare System (VAGLA). Clinical data (e.g., cirrhosis etiology, cirrhosis complications, medications, and clinical laboratory tests) were captured from electronic medical records using standardized case report forms. Written informed consent was obtained from all patients, and the study was approved by the Institutional Review Boards at each of the three institutions.

### 2.2. Duodenal Biopsy Collection and Processing

All participants underwent a standard of care upper gastrointestinal endoscopy screening at their baseline study visit at their recruitment site and were asked to donate four duodenal biopsies (1–2 mm each). The biopsies were collected from the 2nd portion of the duodenum, distal to the Ampulla of Vater, using biopsy forceps. Each biopsy was placed in separate 2 mL cryovials and flash frozen using ethanol and dry ice and stored in −80 °C freezers until testing.

### 2.3. Food Frequency Questionnaire

At the baseline study visit, participants completed a food frequency questionnaire (FFQ) to capture usual diet over the last 90 days, using VioScreen^®^ (Viocare, Inc., Princeton, NJ, USA) a web-based, interactive, and graphical dietary assessment that utilizes over 1200 food images. VioScreen was self-administered using a portable tablet. VioScreen’s dietary analysis utilizes the food and nutrient information from the Nutrition Coordinating Center (NCC) Food and Nutrient Database, and it was developed and is maintained by the NCC at the University of Minnesota. VioScreen has been validated against traditional paper-based FFQs [33].

### 2.4. Microbiome Characterization

ZymoBIOMICS DNA Microprep Kit (Zymo Research, Irvine, CA, USA) was used for DNA extraction from the biopsy specimens, per the manufacturer’s protocol. PCR amplification of the V4 region of the 16S ribosomal RNA gene was followed by 250 × 2 paired-end sequencing on an Illumina HiSeq (Illumina, San Diego, CA, USA), as previously described [32,34]. The sequences were processed using the DADA2 pipeline in R, and SILVA 132 database was used for taxonomy assignment [35]. Next, data were incorporated into QIIME 2 version 2019.10 [36]. Amplicon sequence variants were filtered out if they were not present in at least 15% of all samples (3,449,114/4,777,389 sequences were kept after this filtering step). Sequence depths ranged between 9,030 and 246,235 per sample with a median value of 71,618.

### 2.5. Statistical Analysis

For each participant, we calculated the Healthy Eating Index 2010 (HEI-2010) as a measure of diet quality. This instrument assesses conformance with 2010 U.S. federal dietary guidance, the basis for nutrition policy in the U.S. [37]. The HEI-2010 is made up of 12 components that include 9 adequacy components and 3 moderation components. The 12 components were scored for a total of 0 (nonadherence) to 100 (optimal adherence) points. For the adequacy components, participants with a minimum intake that met the dietary recommendation received higher scores. The components included total fruit, whole fruits, total vegetables, greens and beans, total protein foods, seafood and plant proteins, whole grains, dairy, and fatty acids. The last three components were weighted more heavily. For the moderation components, which included refined grains, sodium, and empty calories, intakes at the level of the standard or lower received higher scores, with the most weight given to empty calories. HEI-2010 was analyzed in tertiles of scores over all participants. Additionally, two of the HEI component scores, total protein and whole grains, were analyzed individually in tertiles. For the final presentation, the mid and low tertiles of protein were combined into a single category, because 53% of participants had the highest score (5) for protein adequacy, skewing the distribution of subjects across tertiles. Variables capturing protein categories, animal protein and vegetable protein, were also examined as well as a combined variable for the animal:vegetable protein ratio. Fiber was examined according to compliance with the USDA dietary guidelines of 14 g/kcal [38]. Coffee frequency was examined according to categories of ≥5 cups/week versus <5 cups/week.

Microbiome data were analyzed for alpha diversity, beta diversity, and association of taxa abundance with diet quality and components. Alpha diversity refers to metrics of diversity within a community (i.e., patient sample), which includes the total number of species (richness) and how evenly distributed the members of a community are among the species present (evenness) [39]. Alpha diversity was calculated using the Shannon index (a metric of evenness and richness) with data rarefied to 9,029 sequences. The significance of differences in alpha diversity was calculated by analysis of variance. Beta diversity refers to comparison of microbial composition across communities (i.e., patient samples) based upon which species are present/absent or their relative abundances [40]. Beta diversity was calculated using the DEICODE plugin in QIIME 2 which employs a robust Aitchison distance metric. This newer form of beta diversity metric accounts for the sparse compositional nature of microbiome data and has been shown to yield higher discriminatory power when compared to other used metrics such as UniFrac or Bray–Curtis [41]. Statistical significance of differences in beta-diversity was assessed using a permutation multivariate analysis of variance (adonis package in R). Differential abundance and association of microbial genera with diet features were evaluated using DESeq2 in R, which employs an empirical Bayesian approach to shrink dispersion and fit non-rarified count data to a negative binomial model [42]. *p*-values for differential abundance were converted to *q*-values to correct for multiple hypothesis testing (<0.05 for significance) [43]. We examined a few key variables for their potential influence on the association between diet and microbiome including collection site (CSMC, UCLA, or VAGLA), rifaximin use, and antibiotic use.

Metagenomic data were predicted from the 16S rRNA sequencing data using PICRUSt2 implemented in QIIME2 using default parameters [44]. The DEICODE plugin in QIIME2 was used to create the dissimilarity matrix and ordination was performed by principal coordinate analysis. Significance of differences in predicted metagenomics profiles was assessed using the adonis package in R. Differential abundance testing of predicted bacterial genes was performed using DESeq2 with *p*-values adjusted for multiple hypothesis testing.

## 3. Results

At the time of this study, baseline FFQs were completed by 31% of participants enrolled in the M_3_LD study. We assessed differences in demographic and clinical factors between participants who completed the FFQ (n = 51) and those who did not (N = 114) (Table S1). Participants who completed the FFQ were more likely to be female and not Hispanic or Latino. There were no substantial differences between groups in age, race, cirrhosis etiology, cirrhosis complications, or baseline clinical labs.

Participants who completed the FFQ had a mean age of 57 years, 57% were male, 88% were white, and 27% were Hispanic or Latino (Table 1). The most common cirrhosis etiology was hepatitis B or C virus (HCV/HBV, 31%), followed by alcoholic liver disease (ALD, 24%), and nonalcoholic steatohepatitis (NASH, 16%). Most participants had a history of decompensation, most commonly esophageal varices (73%), ascites (53%), and hepatic encephalopathy (HE, 20%), with an average model for end stage liver disease (MELD) of 11.5. Rifaximin was a baseline medication in 14% of participants, and 12% of participants used other antibiotics at baseline. We compared participant characteristics by tertiles of HEI-2010 score as an overall measure of diet quality. Female sex and older age were associated with higher HEI-2010 scores. Hispanic ethnicity, HCV infection, HE, and a high MELD were associated with lower HEI-2010 scores. We also examined participant characteristics according to protein, fiber, and coffee intake (Table S2). Participants with HE tended to consume a lower amount of protein and higher amount of fiber, and female participants consumed more fiber compared to male participants.

**Figure 1:**
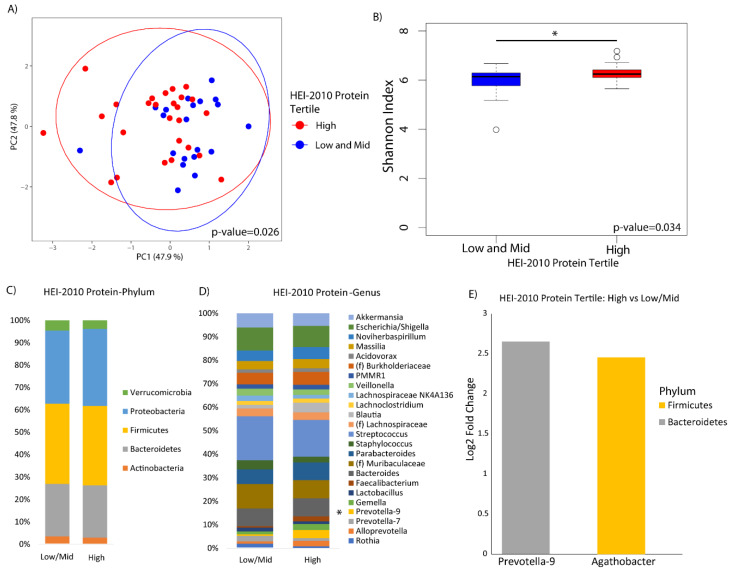
Duodenal microbiome of cirrhotic patients varies by dietary protein. (**A**) Principal coordinate analysis plot of the microbiome colored by high versus mid and low tertiles of protein adequacy according to the Healthy Eating Index-2010 (HEI-2010) and encircled by 99% confidence interval ellipses. (**B**) Box plot of microbial diversity by Shannon index (a metric of richness and evenness) grouped by protein adequacy. (**C**,**D**) Taxonomic summary plots showing the relative abundance of all (**C**) phyla and (**D**) genera (minimum of 1% relative abundance) by protein adequacy. * Represents genera that are differentially abundant (**E**) Log2 fold changes are shown for genera with differential abundance between high versus low protein adequacy in DESeq2 models at *q* < 0.05.

The HEI-2010 overall score was not significantly associated with alpha or beta diversity in this study (Figure S1). When we looked at the HEI protein component individually, participants that had a higher adherence to 2010 U.S. federal dietary guidance for recommended protein intake exhibited a significantly different microbial profile than those with lower adherence based on beta diversity analysis (*p*-value = 0.03), and greater alpha diversity as indicated by the Shannon index (*p*-value = 0.03) (Figure 1A,B). There were no observable differences in bacteria at the phylum level according to protein intake (Figure 1C), however bacteria of the genera *Prevotella-9* and *Agathobacter* exhibited a significant increase in participants who were more adherent compared to those who were less adherent (Figure 1D,E). There was no evidence that animal versus plant protein was associated with different compositional change in the duodenal microbiome.

**Figure 2:**
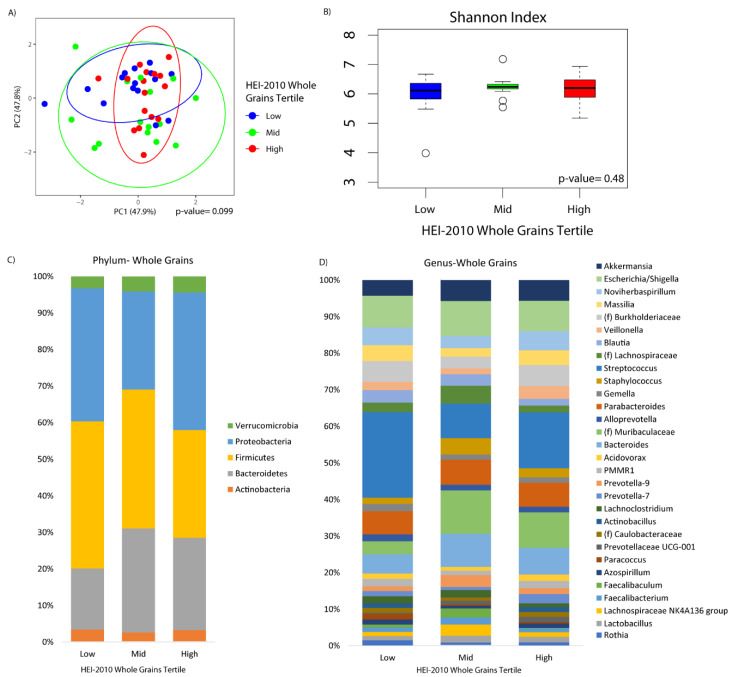
Duodenal microbiome of cirrhotic patients varies by dietary whole grains. (**A**) Principal coordinate analysis plot of the microbiome colored by tertiles of whole grain adequacy according to the HEI-2010, encircled by 99% confidence interval ellipses. (**B**) Box plot of microbial diversity by Shannon Index (a metric of richness and evenness) grouped by whole grain adequacy. (**C**,**D**) Taxonomic summary plots showing the relative abundance of all (**C**) phyla and (**D**) genera (minimum of 1% relative abundance) by tertiles of whole grain adequacy.

**Figure 3:**
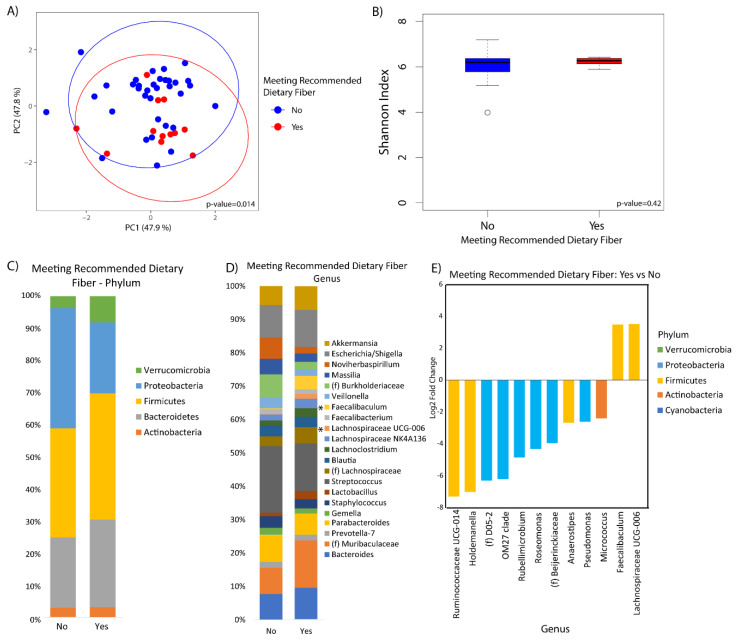
Duodenal microbiome of cirrhotic patients varies by dietary fiber. (**A**) Principal coordinate analysis plot of the microbiome colored by fiber intake based on USDA recommendation of 14 g/kcal encircled by 99% confidence interval ellipses. (**B**) Box plot of microbial diversity by Shannon index (a metric of richness and evenness) grouped by fiber intake. (**C**,**D**) Taxonomic summary plots showing the relative abundance of all (**C**) phyla and (**D**) genera (minimum of 1% relative abundance) by fiber intake. *Represents genera or phyla that are differentially abundant. (**E**) Log2 fold changes are shown for genera with differential abundance between those that met daily dietary fiber intake recommendations versus those that did not in DESeq2 models at *q* < 0.05.

Microbial composition was marginally associated with whole grains consumption (*p*-value = 0.10) (Figure 2A). There was no difference in alpha diversity across the tertiles of whole grains consumption (Figure 2B). At the phylum level, increased Bacteroidetes was observed in the middle and upper tertile as compared to the lower tertile (*q*-value = 0.10 and 0.05, respectively), although there were no observable differences according to whole grain consumption at the genus level (Figure 2C,D). Fiber was significantly associated with beta diversity (*p*-value = 0.01), but not alpha diversity (Figure 3A,B). Compositional changes were also associated with fiber intake (Figure 3C,D), with several taxa significantly increased or decreased in association with fiber (Figure 3E).

**Figure 4:**
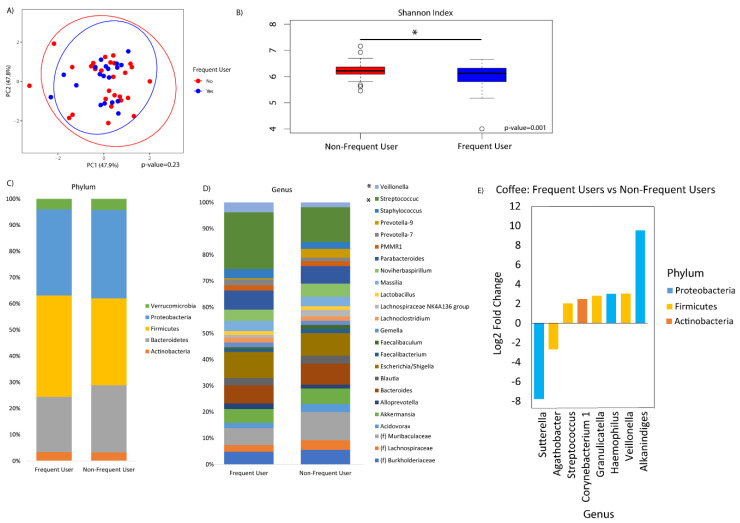
Duodenal microbiome of cirrhotic patients varies by frequency of coffee drinking. (**A**) Principal coordinate analysis plot of the microbiome colored by frequent (≥5 cups/week) versus non-frequent (<5 cups/week) coffee drinking, and encircled by 99% confidence interval ellipses. (**B**) Box plot of microbial diversity by Shannon Index (a metric of richness and evenness) grouped by coffee frequency. (**C**,**D**) Taxonomic summary plots showing the relative abundance of all (**C**) phyla and (**D**) genera (minimum of 1% relative abundance) by frequency of coffee use. (**E**) Log2 fold changes are shown for genera with differential abundance between frequent users versus non-frequent users in DESeq2 models at *q* < 0.05.

**Figure 5:**
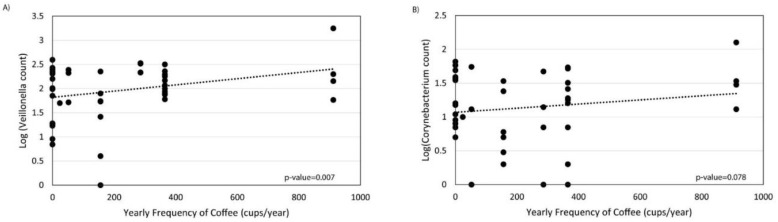
Linear regression of the number of cups of coffee consumed per year by (**A**) *Veillonella* and (**B**) *Corynebacterium 1* relative abundance in the duodenum.

Frequency of coffee drinking was not associated with overall microbial composition (Figure 4A). However, frequent coffee drinkers showed decreased alpha diversity by the Shannon index compared to non-frequent coffee drinkers (*p*-value = 0.001, Figure 4B). There were no significant differences in phylum relative abundances associated with coffee (Figure 4C). At the genus level, there were significant increases in *Streptococcus*, *Corynebacterium_1*, *Granulicatella*, *Haemophilus*, *Veillonella*, and *Alkanindiges* and significant decreases in *Agathobacter* and *Sutterella* associated with frequent coffee drinking (Figure 4D,E). Examining all genera that were significantly different, there was also a dose–response association with coffee drinking and counts of *Veillonella* (*p* = 0.007) and *Corynebacterium_1* (*p* = 0.078) (Figure 5). We did not find that collection site, rifaximin use, or antibiotic use were associated with the duodenal microbiome in this population (data not shown).

**Figure 6:**
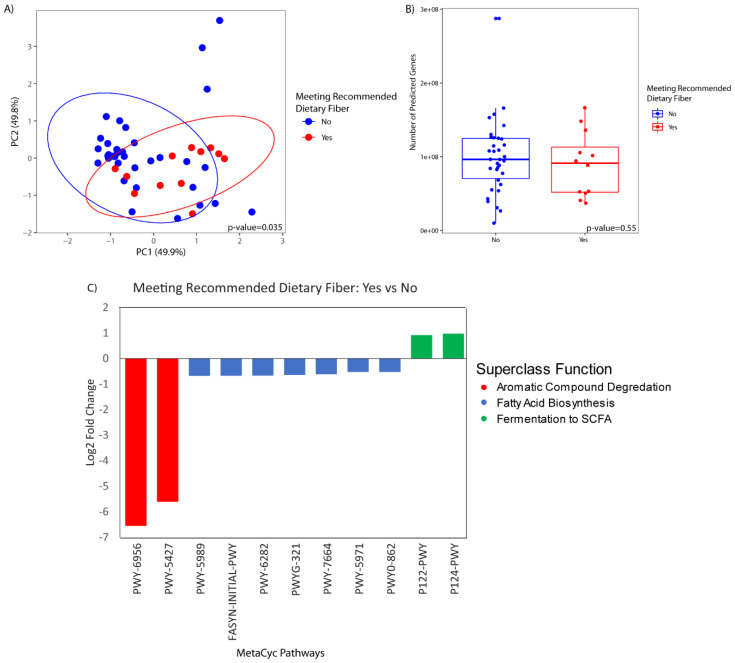
Predicted metagenome grouped by fiber intake based on USDA recommendation of 14 g/kcal. (**A**) Principal coordinate analysis of predicted metagenomic profiles by fiber intake, encircled by 95% confidence interval ellipses. (**B**) Box plot of gene richness (i.e., number of distinct predicted genes) present per sample by fiber intake. Solid bar represents the mean and the box represents 1 standard deviation. (**C**) Differentially abundant functional pathways (*q* < 0.05) in patients that meet the recommended daily fiber intake versus those that do not.

There were no statistically significant differences in the predicted metagenome by HEI-2010, protein, whole grains, or coffee intake. There was a significant difference in the predicted metagenomic profile of participants who met the daily recommended dietary intake of fiber as compared to those that did not (*p*-value = 0.04) (Figure 6A). There was no difference in bacterial gene richness (i.e., the number of predicted genes) between the two groups (Figure 6B). Differential abundance testing demonstrated a significant association of 11 functional pathways with fiber intake. Pathways enriched in participants with higher fiber intake were related to fermentation of short-chain fatty acids, while the pathways that were less abundant in participants with higher fiber intake were related to aromatic compound degradation and fatty acid biosynthesis (Figure 6C).

## 4. Discussion

To our knowledge, this is the first study to report on the association between diet and the microbiome of the proximal gut in patients with liver cirrhosis. Our results suggest that patients with cirrhosis have duodenal microbial profiles that are responsive to underlying dietary practices. We observed differences in the microbiome in relation to total protein, whole grains, fiber, and coffee consumption. There is a growing appreciation that diet quality is an important factor in liver disease progression. Large national cohorts have reported that adherence to a healthful diet, measured using HEI-2010 or other diet quality indices, is associated with a lower risk of chronic liver disease mortality and HCC risk [31,45]. We speculate that the diet’s influence on the microbiome may be a factor in liver disease progression.

Most of the human research on the microbiome and liver disease to date has focused on the fecal microbiome [46]. While stool is considered an adequate representation of the microbiome of the distal gut, it is far less representative of the proximal gut [47]. Anatomically defined segments of the gastrointestinal tract each have distinct physiological roles, immunological components, and microbiomes [48,49]. There is increasing evidence that the small intestinal microbiome is key in harvesting energy from the diet and in maintaining body energy homeostasis, and that nutrient contact in duodenum has powerful metabolic effects, which we believe provides a strong rationale for examining the duodenal microbiome in relation to diet in liver disease [50,51]. Several key factors set the small intestine apart from the colon, including the presence of antimicrobial peptides, greater acidity and oxygenation, more rapid motility, and greater proximity to ingested nutrients [49]. It follows that the small intestinal microbiome is phylogenetically less diverse and less abundant than the colon, yet more dynamic and responsive to environmental factors, such as diet [47]. Thus, given that the duodenum has a distinct microbiome and unique and essential physiological functions for digestion, it is important to characterize its microbiome in relation to nutrition and liver disease.

Although to our knowledge no human studies have examined small intestinal microbiome and diet, a prior experimental study demonstrated that diet can reprogram the small intestinal microbiome in mice [52]. In comparison, numerous studies have shown that changes in fecal microbiota are associated with diet. One prior study examined the association between diet and the fecal microbiome and metabolome in liver cirrhosis, comparing patients in Turkey on a Mediterranean diet to patients in the U.S on a Western diet [30]. This study found that Turkish patients had a more healthful gut microbiome with greater microbial diversity compared to the U.S. patients, an observation that was robust to liver disease status among Turkish patients. In contrast, microbial diversity in the U.S. patients which was lower overall, decreased as severity of liver disease increased. Additionally, several specific dietary components were associated with the microbiome composition in patients with cirrhosis including coffee, tea, vegetables, chocolate, fermented milk, and carbonated beverages. Our study extends this prior research by focusing on the small intestinal microbiome and reports several novel findings.

Protein calorie malnutrition (PCM) is common in liver cirrhosis, affecting between 50% and 90% of patients [23,24], and is associated with an increased risk of morbidity and mortality [25]. PCM pathogenesis is multifactorial and includes impaired protein digestion, absorption, and metabolism, as well as inadequate dietary intake. We found that dietary protein was associated with microbial diversity (alpha and beta diversity) in the duodenum. Our results suggest that disruption of the small intestinal microbiome by low dietary protein intake may be one mechanism contributing to PCM-related liver disease progression, opening the possibility that microbiome may serve as a therapeutic target for this serious cirrhosis-related complication. HE is another serious complication of liver cirrhosis that causes temporary worsening of brain function when the liver cannot properly convert and filter toxins which can cross the blood/brain barrier. Ammonia, a byproduct of amino acid catabolism, is one such toxin and a major cause of HE. Historically, transient protein restriction, which was thought to limit the synthesis of ammonia, was used to treat HE. Later, studies showed that high-protein diets are not only well-tolerated in patients with cirrhosis-related HE but can also improve prognosis [53,54,55]. There is a well-recognized association between gut microbiome and HE. First line therapies for HE includes prebiotics such as lactulose, a sugar that is not absorbed by the digestive tract but works by altering gut microbiota to decrease ammonia production and absorption. In prior research, we found that duodenal microbiome was associated with HE in patients with liver cirrhosis [32], and that *Prevotella-9* (which was increased among patients with protein adequacy) was significantly decreased in patients with HE (unpublished). Taken together, it appears that the microbiome associated with dietary protein may play a role in HE, and this could be more specifically targeted to prevent or treat HE in patients with cirrhosis.

In prior research, *Prevotella* was overrepresented in the small intestinal microbiome of patients with cirrhosis compared to healthy controls [56], and has also been associated with more advanced stages of fibrosis [57]. Increased *Prevotella* abundance has also been associated with increased mucosal inflammation [58]. Diets that are rich in protein tend to be more inflammatory than those diets that are rich in vegetables and fiber, which provides one explanation for our observation that *Prevotella-9* was significantly increased in association with high protein consumption. In other research, *Agathobacter*, which was also increased in association with higher protein intake, was increased in patients receiving a dietary intervention of barley beta glucans [59]. These soluble non-starch polysaccharides have numerous physiological effects, some of which may correlate with a high protein diet [60].

Whole grains are a good source of fiber, vitamins, minerals, and phytonutrients. Increased intake of whole grain has been associated with improved insulin sensitivity, metabolic regulation, and reduced inflammation [61]. Additionally, increased whole grain intake, and fiber specifically, have been associated with a significantly lower risk of HCC [28]. Our finding that whole grain (particularly the middle tertile) and fiber adequacy were associated with the duodenal microbiome, suggests that the benefits of moderate whole grains and adequate fiber in liver disease may be mediated through their effects on the duodenal microbiome. Importantly, we observed an increase in bacteria that degrade complex polysaccharides to short-chain fatty acids (i.e., *Lachnospiraceae*), and an upregulation of genes involved in short-chain fatty acid synthesis, in association with fiber adequacy, suggesting that the duodenal microbiome composition is important for fiber digestion. We found that fiber was associated with beta diversity but not alpha diversity in our study. Fecal microbiome studies have shown that diets high in fiber are associated with increased alpha diversity, yet fiber intervention studies have consistently reported a lack of association between fiber and alpha diversity [62,63,64].

Coffee appears to have a strong effect in lowering the risk of chronic liver disease and HCC [29]. A recent study investigated the mechanisms using untargeted metabolomics and identified several bile acids that were significantly associated with coffee drinking, as well as risk for HCC and death from liver disease [65]. A growing body of literature shows that constituents of coffee, including caffeine and cafestol, have antimicrobial and immunomodulatory effects [66,67,68,69]. *Veillonella*, which had a log-linear association with coffee frequency in our study, has previously been found to be elevated in elite athletes [70]. Experimental studies show that *Veillonella* utilizes lactic acid (an exercise by-product and a key acid component of coffee) as their main food source [70]. Our finding that coffee consumption was associated with the duodenal microbiome composition is compatible with these prior findings and suggests that the duodenal microbiome may be a functional link between coffee drinking and liver disease pathogenesis, possibly through modulation of harmful bile acids.

This unique study reports several novel findings with regards to duodenal microbial composition and diet and in patients with cirrhosis, but there are several limitations. Our decision to focus on the duodenal microbiome precludes direct comparison with prior publications that have characterized the stool microbiome in relation to liver disease. Our justification is based on the putatively important and unique physiological role of the duodenum, and we hope that our observations will inspire future investigations of the small intestinal microbiome in liver disease for which this study can provide a basis for comparison. Although this study focused on dietary practices to assess healthfulness, diet alone may not be an adequate measure of nutritional status in cirrhosis [23]. For example, hypermetabolism due to increased resting energy expenditure, and malabsorption due to portosystemic shunting, are important factors affecting nutritional status in cirrhosis. Thus, future research should be conducted to understand the association between the duodenal microbiome and these and other measures of nutritional status in cirrhosis. Furthermore, while we focused on diet quality and dietary factors selected a priori for their associations with liver disease and cancer, there may be associations with additional macro and micronutrients that we were unable to examine due to the small study sample. Additionally, while the HEI index reflects guidelines for the average American, it may not represent ideal nutritional needs for a patient with liver cirrhosis. For example, according to guidelines, patients with cirrhosis are recommended to consume a greater amount of protein (1.0 to 1.5 g/kg per day protein) than the 0.8 g/kg per day recommended for healthy individuals [71].

In conclusion, we present results on the first known study of the duodenal microbiome and diet in patients with liver cirrhosis. We conclude that protein, fiber, and coffee are associated with diversity and composition of the duodenal microbiome in liver cirrhosis.

## Figures and Tables

**Table 1:** Select characteristics of study participants.

Demographics	Complete	HEI-2010		
		Lowest Tertile	Middle Tertile	Highest Tertile
Number	51	17	17	17
Site, n (%)				
CSMC	24 (47%)	6 (35%)	9 (53%)	9 (53%)
UCLA	26 (51%)	11 (65%)	8 (47%)	7 (41%)
VAGLA	1 (2%)	0	0	1 (6%)
Sex, n (%)				
Male	29 (57%)	11 (65%)	10 (59%)	8 (47%)
Female	22 (43%)	6 (35%)	7 (41%)	9 (53%)
Baseline age, mean (st dev)	57 (11)	52.6 (9.6)	57.2 (11.2)	60.5 (11.6)
Race, n (%)				
White	45 (88%)	14 (82%)	16 (94%)	15 (88%)
Non-White	6 (12%)	3 (18%)	1 (6%)	2 (12%)
Ethnicity, n (%)				
Hispanic or Latino	14 (27%)	7 (41%)	4 (24%)	3 (18%)
Not Hispanic or Latino	37 (73%)	10 (59%)	13 (76%)	14 (82%)
Cirrhosis etiology, n (%)				
HCV/HBV	16 (31%)	7 (41%)	6 (35%)	3 (18%)
ALD	12 (24%)	4 (24%)	4 (24%)	4 (24%)
NASH	8 (16%)	1 (6%)	3 (18%)	4 (24%)
PSC	7 (14%)	3 (18%)	2 (12%)	2 (12%)
Other	8 (16%)	2 (12%)	2 (12%)	4 (24%)
Cirrhosis Complications, n (%)				
Hepatic Encephalopathy	10 (20%)	1 (6%)	6 (35%)	3 (18%)
Esophageal Varices	37 (73%)	10 (59%)	13 (76%)	14 (82%)
Ascites	27 (53%)	9 (53%)	10 (59%)	8 (47%)
Baseline clinical labs, mean (st dev)				
AFP	4.7 (4.3)	4.8 (3.7)	5.4 (6.1)	3.8 (1.7)
Creatinine	1 (0.9)	1.3 (1.4)	1.1 (0.45)	0.74 (.20)
Bilirubin	1.8 (2.0)	1.8 (1.8)	1.7 (1.5)	1.9 (2.6)
AST	42 (23)	42 (19)	45 (28)	39 (21)
ALT	33 (18)	36 (18)	32 (19)	30 (17)
Platelets	125 (78)	125(62)	138 (88)	112 (86)
INR	1.2 (0.2)	1.2 (0.22)	1.2 (0.11)	1.3 (0.31)
MELD	11.5 (5.7)	12 (6.5)	11 (4.7)	11 (5.9)
Baseline medications, n (%)				
PPI	23 (45%)	6 (35%)	10 (59%)	7 (41%)
Lactulose	9 (18%)	1 (6%)	3 (18%)	5 (29%)
Rifaximin	7 (14%)	0	3 (18%)	4 (24%)
Antibiotics	6 (12%)	3 (18%)	0	3 (18%)

Abbreviations: HEI, healthy eating index; CSMC, Cedars-Sinai Medical Center; UCLA, Ronald Reagan UCLA Medical Center; VAGLA, Veterans Affairs Greater Los Angeles Healthcare System. HCV, hepatitis C virus; HBV, hepatitis B virus; ALD, alcoholic liver disease; NASH, non-alcoholic steatohepatitis; AIH, autoimmune hepatitis; PBC, primary biliary cirrhosis; PSC, primary sclerosing cholangitis; AFP, alpha-fetoprotein; AST, aspartate aminotransferase; ALT, alanine aminotransferase; INR, international normalized ratio; MELD, model for end stage liver disease; PPI, proton pump inhibitor.

## Supplementary Materials

The following are available online at https://www.mdpi.com/2072-6643/12/5/1395/s1, Figure S1: (**A**) Principal coordinate analysis plot of the microbiome colored by HEI2010 score tertiles and encircled by 99% confidence interval ellipses. (**B**) Box plot of microbial diversity by Shannon Index (richness/evenness) grouped by HEI2010 score tertiles, Table S1: Comparison of characteristics for participants with and without diet information, Table S2: Select characteristics of study participants by protein, fiber, and coffee intake.
