# Supplementary material for: Dietary Protein, Fiber and Coffee Are Associated with Small Intestine Microbiome Composition and Diversity in Patients with Liver Cirrhosis
Source: Nutrients. 2020 May 13;12(5):1395. doi: 10.3390/nu12051395 (PMC7285216; doi:10.3390/nu12051395)
Supplement: Supplementary file 1 [file nutrients-12-01395-s001.pdf]

Supplementary Table 1. Comparison of participant characteristics between those with and without diet information

**Demographics**	**Microbiome-only**	**Microbiome-diet**
Number	114	51
Sex, n (%)		
Male	79 (69%)	29 (57%)
Female	35 (31%)	22 (43%)
Baseline age, mean (st dev)	59 (13)	57 (11)
Race, n (%)		
American Indian/Alaskan native	3 (3%)	0
Asian	5 (4%)	2 (4%)
Black of African American	11 (10%)	3 (6%)
More than one race	2 (2%)	1 (2%)
White	93 (82%)	45 (88%)
Ethnicity, n (%)		
Hispanic or Latino	42 (37%)	14 (27%)
Not Hispanic or Latino	72 (63%)	37 (73%)
Cirrhosis etiology, n (%)		
HCV	42 (37%)	15 (29%)
HBV	2 (2%)	1 (2%)
ALD	30 (26%)	12 (24%)
NASH	20 (18%)	8 (16%)
AIH	10 (9%)	3 (6%)
PBC	1 (1%)	2 (4%)
PSC	0	7 (14%)
Other	9 (8%)	3 (6%)
Cirrhosis Complications, n (%)		
Hepatic Encephalopathy	42 (30%)	10 (20%)
Esophageal Varices	60 (54%)	37 (73%)
Ascites	49 (44%)	27 (53%)
Baseline clinical labs, mean (st dev)		
AFP	9.8 (35.0)	4.7 (4.3)
AFP <20ng/mL (%), n (%)	78 (96%)	41 (98%)
AFP 20-400 ng/mL (%), n (%)	3 (4%)	1 (2%)
Creatinine	1.1 (1.1)	1 (0.9)
Sodium	138 (3.6)	138 (3.7)
Albumin	3.8 (0.7)	3.7 (0.7)
Bilirubin	1.5 (2.0)	1.8 (2.0)
ALP	130 (122)	136 (74)
AST	51 (55)	42 (23)
ALT	41 (51)	33 (18)
Platelets	125 (67)	125 (78)
INR	1.2 (0.2)	1.2 (0.2)

PT	13.7 (3.0)	13 (3.0)
MELD	10.9 (5.4)	11.5 (5.7)
Baseline medications, n (%)		
PPI	47 (41%)	23 (45%)
Lactulose	29 (25%)	9 (18%)
Rifaximin	21 (18%)	7 (14%)

Supplementary Table 2. Select characteristics of study participants by protein, fiber, and coffee intake

	**Protein**[1]		**Fiber**[2]		**Coffee**[3]	
**Demographics**	**Low and mid**	**High**	**Not meeting recommendations**	**Meeting recommendations**	**Not frequent**	**Frequent**
Number	24	27	36	15	29	22
Sex, n (%)						
Male	14 (58%)	15 (56%)	22 (61%)	7 (47%)	17 (59%)	12 (55%)
Female	10 (42%)	12 (44%)	14 (39%)	8 (53%)	12 (41%)	10 (45%)
Baseline age, mean (st dev)	54 (13)	59 (9)	56 (12)	59 (9)	53 (12)	62 (8)
Race, n (%)						
White	21 (88%)	24 (89%)	33 (92%)	12 (80%)	27 (93%)	18 (82%)
Non-White	3 (12%)	3 (11%)	3 (8%)	3 (20%)	2 (7%)	4 (18%)
Ethnicity, n (%)						
Hispanic or Latino	7 (29%)	7 (26%)	11 (31%)	3 (20%)	7 (24%)	7 (32%)
Not Hispanic or Latino	17 (71%)	20 (74%)	25 (69%)	12 (80%)	22 (76%)	15 (68%)
Cirrhosis etiology, n (%)						
HCV/HBV	6 (25%)	10 (37%)	12 (33%)	4 (27%)	8 (28%)	8 (36%)
ALD	7 (29%)	5 (19%)	9 (25%)	3 (20%)	8 (28%)	4 (18%)
NASH	2 (8%)	6 (22%)	5 (14%)	3 (20%)	2 (7%)	6 (27%)
PSC	4 (17%)	3 (11%)	4 (11%)	3 (20%)	6 (21%)	1 (5%)
Other	5 (21%)	3 (11%)	6 (17%)	2 (13%)	5 (16%)	3 (14%)
Cirrhosis Complications, n (%)						
Hepatic Encephalopathy	7 (29%)	3 (11%)	6 (17%)	4 (27%)	6 (21%)	4 (18%)
Esophageal Varices	20 (83%)	17 (63%)	27 (75%)	10 (67%)	21 (72%)	16 (73%)
Ascites	14 (58%)	13 (48%)	20 (56%)	7 (47%)	16 (55%)	11 (50%)

Abbreviations: HEI, healthy eating index; HCV, hepatitis C virus; HBV, hepatitis B virus; ALD, alcoholic liver disease; NASH, non-alcoholic steatohepatitis; AIH, autoimmune hepatitis; PBC, primary biliary cirrhosis; PSC, primary sclerosing cholangitis; [1]Protein (low and mid=lowest and middle tertiles based on HEI-2010, high=highest tertile based on the HEI-2010); [2]Fiber adequacy is based on USDA recommendation of 14 grams/kcal; [3]Not frequent coffee intake is < 5 cups/week, frequent coffee intake is ≥ 5 cups/week;

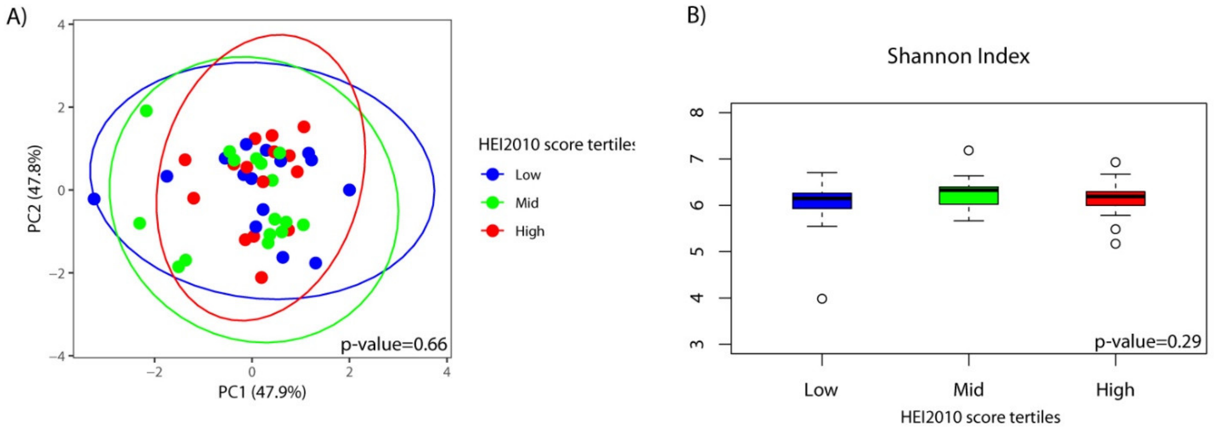

Supplementary Figure 1: (A) Principal coordinate analysis plot of the microbiome colored by HEI2010 score tertiles and encircled by 99% confidence interval ellipses. (B) Box plot of microbial diversity by Shannon Index (richness/evenness) grouped by HEI2010 score tertiles.
